# A válvula aórtica bicúspide e desordens relacionadas

**DOI:** 10.1590/S1516-31802010000500010

**Published:** 2010-09-02

**Authors:** Shi-Min Yuan, Hua Jing

**Affiliations:** I MD, PhD. Postdoctoral researcher, Department of Cardiothoracic Surgery, Jinling Hospital, School of Clinical Medicine, Nanjing University, Nanjing 210002, Jiangsu Province, People’s Republic of China.; II MD. Professor and head, Department of Cardiothoracic Surgery, Jinling Hospital, School of Clinical Medicine, Nanjing University, Nanjing 210002, Jiangsu Province, People’s Republic of China.

**Keywords:** Aorta, thoracic, Aortic valve, Coronary artery disease, Heart defects, congenital, Endocarditis, Aorta torácica, Valva aórtica, Doença da artéria coronariana, Cardiopatias congênitas, Endocardite

## Abstract

Bicuspid aortic valve (BAV) is the most common congenital cardiac malformation, affecting 1-2% of the population, with strong male predominance. Individuals may have a normally functioning BAV, and may be unaware of its presence and the potential risk of complications. However, they may easily develop aortic valve disorders: either stenotic or regurgitant, or both. Today, BAV is recognized as a syndrome incorporating aortic valve disorders and aortic wall abnormalities, including aortic dilation, dissection or rupture. Congenital or hereditary diseases such as ventricular septal defect, patent ductus arteriosus, coarctation of the aorta, Turner’s syndrome, Marfan’s syndrome etc., may frequently be associated with BAV. Infective endocarditis and occasionally thrombus formation may develop during the lives of BAV patients. Elevated cholesterol or C-reactive protein may be seen in laboratory findings of these patients. Beta-blockers and statins are the possibilities for medical treatment, and aortic valve repair/replacement and ascending aorta replacement are indicated for patients with a severely diseased aortic valve and aorta. Rigorous follow-up throughout life is mandatory after BAV has been diagnosed. The aim of the present article was to describe the implications of BAV and its associated disorders, and to discuss diagnostic and treatment strategies.

## RESUMO

A válvula aórtica bicúspide (VAB) é a malformação cardíaca congênita, mais comum afetando 1-2% da população, com forte predominância no sexo masculino. Indivíduos afetados podem exibir funcionamento normal da VAB, ignorando sua presença e o potencial risco de complicações associadas. Mas podem facilmente desenvolver disfunções de valva aórtica, com estenose, regurgitação, ou ambas. Hoje em dia, a VAB é reconhecida como uma síndrome incorporando alterações de valva aórtica e anormalidades da parede aórtica, inclusive dilação da aorta, dissecção ou ruptura. Doenças congênitas ou hereditárias, como defeito do septo ventricular, canal arterial patente, coartação de aorta, síndrome de Turner, síndrome de Marfan etc., podem estar frequentemente associadas com VAB. A endocardite infecciosa e ocasionalmente formação de trombo podem se desenvolver durante a vida nos pacientes com VAB. Elevação do colesterol ou proteína C-reativa pode ser observada nos resultados laboratoriais desses pacientes. Betabloqueador e estatinas são as opções de tratamento médico e reparação/substituição da valva aórtica e substituição da aorta ascendente são indicadas para pacientes com doença grave da valva aórtica e artéria aorta. Acompanhamento rigoroso por toda a vida é obrigatório quando do diagnóstico da VAB. O objetivo do presente artigo é descrever as implicações do VAB assim como suas desordens associadas e discutir as estratégias de diagnóstico e tratamento.

## INTRODUCTION

Bicuspid aortic valve (BAV) is the most common congenital cardiac malformation, affecting 1-2% of the population, with strong male predominance.^1^ It may occur in isolation, or in association with other congenital heart diseases.^2^ Individuals may have a normally functioning BAV, and may be unaware of its presence and the potential risk of impending complications.^3^ They may typically remain asymptomatic until the third or fourth decade of life, when the valve becomes dysfunctional. They then require close follow-up, and valve replacement may be warranted.^4^ BAV can be associated with abnormalities of the aortic wall such as coarctation of the aorta, aortic dissection, aortic aneurysm and Turner’s syndrome.^4^ It is now accepted that BAV is associated with both valve disease and aortic disease,^5^ thereby leading to increased morbidity and mortality, including aortic valve disorders, aortic wall abnormalities, endocarditis and other cardiovascular malformations.^6^ BAV has been identified at a prevalence of 4.6 cases per 1000 live births. The prevalence of BAV according to sex has been found to be 7.1 cases per 1000 among male neonates, and 1.9 per 1000 among female neonates. All newborns with BAV are asymptomatic. Mild aortic regurgitation has only been found in one neonate with BAV.^6^

## ETIOLOGY

It has been noted that BAV is inheritable. Families presenting familial congenital BAV have been described.^7^ A patient with aneurysm of the ascending aorta and calcific stenosis of a BAV, whose brother also had a stenotic BAV, has been reported.^8^

The pathogenesis of BAV is unknown. Experiments on Syrian hamsters have revealed that BAV does not occur consequent to improper development of the conotruncal ridges, conotruncal malseptation, valve cushion agenesis, or lesions acquired after normal valvulogenesis. Fusion of the right and left valve cushions at the beginning of valvulogenesis appears to be a key factor in BAV formation.^9^ A recent study has demonstrated that BAVs with fused right and noncoronary leaflets and those with fused right and left leaflets are different etiological entities. BAVs with fused right and noncoronary leaflets result from a morphogenetic defect that occurs before cardiac outflow tract septation on the basis of an exacerbated nitric oxide-dependent epithelial-to-mesenchymal transformation. On the other hand, BAVs with fused right and left leaflets result from anomalous septation of the proximal portion of the cardiac outflow tract, caused by dysfunctional neural crest cells.^10^ Deficient fibrillin-1 content in the vasculature of BAV patients may trigger matrix metalloproteinase production, thereby leading to matrix disruption and dilation.^11^

It has been noted that the fibrillin-1 content was remarkably reduced in the aorta of BAV patients, compared with that of patients with a tricuspid aortic valve.^12-14^

## CORONARY ARTERY DISORDERS

The incidence of left dominance in BAVs has been found to be unusually high (24.4-56.8%), compared with the incidence in tricuspid valves (9.5%).^15-18^ Patients with BAVs have higher incidence of immediate bifurcation of the left main coronary artery, and higher incidence of left main coronary length less than 10 mm. The mean length of the left main coronary artery is significantly shorter in BAV patients. These variations from the usual coronary artery anatomy may be part of the developmental abnormalities responsible for BAVs.^19^ Anomalous origins of the right^20,21^ and left^22^ coronary arteries, association with annuloaortic ectasia, and anomalous origins of the left circumflex coronary artery^23^ and single left coronary artery,^24^ have been noted in patients with BAVs. Spontaneous coronary artery dissection may occur in BAV patients.^25^

## AORTIC VALVE DISORDERS

BAVs may progress and become calcified, thus leading to varying degrees of severity of aortic stenosis or aortic regurgitation, or both, which may eventually necessitate surgical intervention.^26^ BAV is recognized as a frequent cause of aortic stenosis in adults. Aortic stenosis has been found in 72% of adults with BAV. The stenotic valves were obstructed by nodular, calcareous masses, but commissural fusion was present in only eight cases. Primary aortic regurgitation without infective endocarditis was uncommon, and 32% had an apparently normally functioning aortic valve.^27^ Among the 600 patients analyzed, 213 (36%) had pure aortic stenosis, 265 (44%) had pure aortic regurgitation and 122 (20%) had combined stenosis and regurgitation. BAVs represented 18%, as the third most important cause of aortic disorder following degenerative and rheumatic changes, followed by infective endocarditis (5%).^28^ In 388 patients with severe aortic valve disease alone, BAVs were found in 45% of the patients with aortic stenosis and 24% of the patients with aortic regurgitation. In 110 patients with severe combined aortic and mitral valve disease, BAVs were found in only 12%.^29^ A double-blind placebo-controlled study illustrated that the patients recruited into the ASTRONOMER study were younger, with less severe aortic stenosis. The population of BAV patients was large and accounted for 48.9%.^30^ From echocardiography, the patients with a stenotic BAV had significantly larger anatomical aortic valve areas than effective aortic valve areas. The discrepancy relating to jet eccentricity was much bigger than that of the patients with a stenotic tricuspid aortic valve, thus indicating greater severity of valve dysfunctional hemodynamics. In other words, the jet eccentricity correlated with BAV.^31^

## AORTIC WALL ABNORMALITIES

BAV patients tend to develop vascular abnormalities of the aorta, such as dilation, coarctation and dissection. Aortic dilation in BAV patients is thought to be caused by intrinsic aortic disease that is characterized by cystic medial necrosis and disruption of the extracellular matrix due to fibrillin deficiency.^32^ There is controversy in the literature regarding whether aortic dilation, aneurysm and dissection are hemodynamic complications of BAV or are associated changes. BAV is associated with accelerated degeneration of the aortic media, thus indicating that BAV disease is a pathological process, not a developmental event.^11^

In the ascending aorta as well as the pulmonary trunk, the severity of cystic medial necrosis, elastic fragmentation and changes in the smooth muscle cell orientation have been found to be significantly more severe in patients with bicuspid valves than in those with tricuspid valves.^33^ Factors leading to aortic dissection four years after the Bentall operation have been considered to be an impact of congenital BAV or proximal anastomosis of venous grafts, or both.^34^

Half of young patients with normally functioning BAVs have echocardiographic evidence of aortic dilation. Aortic dilation is believed to be a precursor of aortic rupture and dissection.^11^ Aortic dilation was found to be present in 83.2% of a group of patients, and 83.7% of the cases were in the middle of the ascending tract.^35^ Aortic stenosis and hypertension were the most significant predictors of mid-ascending aneurysm.^36^ Ninety-four percent of aortic dilations associated with normally functioning BAVs showed the same anatomical configuration, with dilation of the mid-ascending tract but normal root diameters, compared with 95% of dilations associated with stenotic tricuspid aortic valves.^37^ The diameter of the ascending aorta in patients with BAV and dilation was significantly larger than in those with a tricuspid aortic valve and dilation. The distance between the aortic valve level and the point of maximum diameter of the ascending aorta in BAV patients without dilation was greater than in coronary patients. All patients with BAV and enlargement of the ascending aorta showed asymmetric dilation of the vessel.^38^

Studies have suggested that patients with BAV have an intrinsic defect in the aortic wall that results in aortic disease, regardless of aortic valve function.^39^ BAV was associated with significantly less intimal change, and less fragmentation and loss of elastic tissue, compared with patients with a tricuspid aortic valve.^40^ Type I and III collagens were significantly decreased in dilated aortas of BAV patients, compared with controls, particularly at the convexity. Expression of messenger RNA (ribonucleic acid) for collagens was lower than normal only in the regurgitant subgroup. Fewer smooth muscle cells and greater severity of elastic fiber fragmentation were observed at the convexity than at the concavity.^41^

BAV, aortic coarctation and systemic hypertension are established risk factors for aortic dissection in the general population and they often occur in Turner’s syndrome.^42^ Approximately 25-30% of women with Turner’s syndrome have a cardiovascular malformation, and in Turner’s syndrome there appears to be a confluence of established risk factors, leading to aortic dissection.^43,44^ Among 119 cases of fatal dissecting aneurysm of the aorta, 11 cases of congenital BAV (9%) were observed. Among the latter, three had coarctation of the aorta and one had Turner’s syndrome without coarctation. In each case, cystic medial necrosis of the aorta was present. Hypertension was either established or inferred from cardiac weight in 73% of the cases. The high incidence among subjects with dissecting aneurysm suggested a causative relationship between BAV and aortic dissecting aneurysm.^45^

Pathological examination of surgical specimens from the aortic wall of patients with aortic dissection associated with BAV showed cystic medial necrosis or mucoid degeneration.^46^ Patients with BAV had thinner elastic lamellae of the aortic media and greater distances between the elastic lamellae than did patients with a tricuspid aortic valve.^47^ Matrix metalloproteinases (endogenous enzymes that degrade matrix components) have been implicated in atherosclerotic aortic aneurysm formation and appear to be elevated in the aorta of patients with BAVs.^11^

The association between Marfan’s syndrome and BAVs is uncommon.^48^ However, many previous studies have outlined similarities between Marfan’s syndrome and BAV. Both are prone to disruption of the aortic wall. In BAV patients, the impairment of aortic elasticity may result from abnormal elastic fibers in the degenerated aortic medial layer.^49^

## CONGENITAL HEART DEFECTS

Patent ductus arteriosus and ventricular septal defect are the most frequent congenital heart defects associated with BAV.^50,51^ There is significantly higher incidence of aortic arch obstruction (51.1%).^52^ No significant relationship between the occurrence of BAVs and left ventricular outflow tract obstructions or mitral valve malformations have been noted. Concurrence of BAVs and bicuspid pulmonary valves has been detected in one study, and some cases presented trisomy#18. The frequency of BAV in specimens with complete transposition of great arteries has been found to be only 1%.^53^ Hypoplastic left heart syndrome, complete atrioventricular canal defect, Ebstein’s anomaly, partial or total anomalous pulmonary venous return, tetralogy of Fallot, double-outlet right ventricle,^54^ septal left ventricular diverticulum,^55^ Williams syndrome,^56^ Down syndrome^57^ and annuloaortic ectasia^58^ are occasionally associated with BAV. Shone’s complex, which is defined by four cardiovascular defects including supravalvular mitral membrane, valvular mitral stenosis with a parachute mitral valve, subaortic stenosis and aortic coarctation, is a rare entity and forms another association in BAV cases.^59^

## INFECTIVE ENDOCARDITIS

Most patients with BAV will develop some complications during their lives. The most common and severe of these is infective endocarditis. Bayer et al.^60^ reported that BAV endocarditis represented only two out of 59 cases in native heart valves. The usual prevalence of infective endocarditis of BAVs is 10% to 16%.^61^ Most patients are unaware of their condition until the onset of infective endocarditis.^62^

Patients with BAV endocarditis are young, and there is strong male predominance. Staphylococci and viridans streptococci account for nearly three-quarters of the cases affecting BAVs.^61^ Infective endocarditis in BAV patients may occur with aortic regurgitation and heart failure at an early age. Many of these patients need surgical intervention, with poor results. Endocarditis vegetation, perforation and perivalvular abscess may occur.^62^

## THROMBUS FORMATION

Thrombus formation in a native BAV is a rare complicaton.^63^ Pathological studies have indicated that post-inflammatory changes occur in the resected BAV, which is prone to develop thrombosis on the valve surface or in the calcification area.^64^ Microthrombus formation and valve thickening with incompetence could result in embolization, and subsequent cerebrovascular events.^65^ Embolization from calcific BAVs may lead to stroke and myocardial infarction. Conservative management with anticoagulation, to treat associated post-stagnation thrombosis, or aortic valve replacement as the treatment, is debatable.^66^ Sudden death may occur as a result of obstruction of the left ventricular outflow tract by a congenital BAV.^67^

## LABORATORY WORKUP

BAV patients often have high levels of cholesterol and C-reactive protein. High serum cholesterol levels have been associated with massive calcification in BAV patients.^68^ C-reactive protein levels have been found to be higher among patients with moderate-to-severe valvular calcification than among patients without or with minimal valvular calcification. Patients with aortic stenosis may have higher C-reactive protein levels than the levels in patients with aortic regurgitation. The C-reactive protein levels in BAV patients have not been found to correlate with aortic diameter, but have shown an association with advanced calcific valve disease.^69^ The plasma levels of C-reactive protein have been found to be higher in patients with BAVs or degenerative stenotic tricuspid aortic valves than in normal controls and patients with pure aortic regurgitation.^70^ In the presence of aortic dilation or infective endocarditis, patients may have anemia with an elevated erythrocyte sedimentation rate.^59^

## DIAGNOSIS AND TREATMENT

The tests that may show a bicuspid aortic valve include magnetic resonance imaging of the heart and echocardiograms, which are sensitive enough to identify the presence or absence of BAVs.

Currently, the treatment for BAV is primarily surgical. Different surgical options exist for BAV patients, depending on the age at presentation and the size and appearance of the aorta.^26^ Patients with BAV should undergo elective repair of the aortic root or replacement of the ascending aorta if the diameter of these structures exceeds 5.0 cm.^71^ Valve-sparing techniques, sometimes with pericardial leaflet extensions, may be the first choice.^72,73^ Triangular resection of the prolapsing larger cusp including the middle raphe, with or without a complementary subcommissural annuloplasty, may lead to satisfactory surgical outcomes.^74^ Aortic valve replacement is indicated for severe aortic stenosis or aortic insufficiency/regurgitation in symptomatic patients, or in those with abnormal left ventricular function. Peak gradient ≥ 80 mmHg and aortic valve area ≤ 0.75 cm^2^ have been found to be predictive of aortic valve replacement for BAV patients.^75^ In neonates, critically stenotic BAVs can be relieved successfully, with a dramatic decrease in left ventricular pressure, through dilation by means of retrograde aortic balloon valvuloplasty.^76^

Triangular resection of the enlarged leaflet has been found to result in optimal valve function, thus leading to normal ventricular dimensions and functions after long-term follow-up.^77^ Reconstruction of incompetent BAVs may significantly reduce left ventricular volume load.^78^ Patients with severe left ventricular outflow tract obstruction who underwent the Ross-Konno procedure had a survival of 98.5% after a median follow-up of 2.4 years.^79^ Schmidtke et al.^80^ recommended that the subcoronary Ross procedure with pulmonary autograft for BAV patients should be used because of the stable postoperative root dimensions seen after long-term follow-up.

Patients with bicuspid valves should uniformly be advised to adhere to antibiotic prophylactics.^1^ It has been suggested that beta-blocker medications can reduce the progression of ascending aortic aneurysms, although the benefit of beta-blockers for preventing aortic dilation in BAV disease is unclear. Nonetheless, hypertension should be carefully monitored and controlled.^81^ Statin medications are useful for preventing calcium buildup on the aortic valve, and for reducing the risk of aortic stenosis.^26^

A literature search was made in Lilacs, PubMed, Embase and the Cochrane Library using the search term "bicuspid aortic valve". A manual search of abstracts of articles was made to identify those relating to the topic. The results are presented in Table 1.

**Table 1.** Results from literature search in the major medical databases

Database	Search strategies	Results		
Cochrane	Bicuspid aortic valve	5 found	4 related	4 randomized controlled trials
Embase (Excerpta Medica database) (1983-2010)		118 found	114 related	28 case reports 15 prospective cohort studies 10 prospective comparative studies 8 research studies 8 retrospective cohort studies 4 retrospective comparative studies 9 reviews 32 others
Lilacs (Literatura Latino-Americana e do Caribe em Ciências da Saúde)		104 found	9 related	3 case reports 5 clinical trials 1 review
PubMed		1401 found	1160 related	12 case control studies 440 case reports 41 prospective comparative studies 73 prospective cohort studies 3 randomized controlled trials 47 retrospective comparative studies 289 retrospective cohort studies 119 research studies 83 reviews 53 others

## CONCLUSIONS

BAV has been recognized as a syndrome incorporating aortic valve disorders and aortic wall abnormalities. Congenital or hereditary diseases such as ventricular septal defect, patent ductus arteriosus, coarctation of the aorta, Turner’s syndrome, Marfan’s syndrome etc. may frequently be associated with BAV. Infective endocarditis and occasionally thrombus formation may develop during the lives of BAV patients. BAV and its associated disorders and possible complications place patients at higher risk. Regular follow-up becomes mandatory after BAV has been diagnosed, in order to closely observe such patients with regard to progression of the disease itself and its complications, and in order to suggest treatments. Beta-blockers and statins are the possibilities for medical treatment, and aortic valve repair/replacement and ascending aorta replacement are indicated for patients with a severely diseased aortic valve and aorta.
